# *Yersinia pseudotuberculosis* BarA-UvrY Two-Component Regulatory System Represses Biofilms via CsrB

**DOI:** 10.3389/fcimb.2018.00323

**Published:** 2018-09-18

**Authors:** Jeffrey K. Schachterle, Ryan M. Stewart, M. Brett Schachterle, Joshua T. Calder, Huan Kang, John T. Prince, David L. Erickson

**Affiliations:** ^1^Department of Microbiology and Molecular Biology, Brigham Young University, Provo, UT, United States; ^2^Department of Chemistry and Biochemistry, Brigham Young University, Provo, UT, United States

**Keywords:** *Yersinia pseudotuberculolisis*, biofilm, carbon storage regulator system A (CsrA), two-component regulation, fleas

## Abstract

The formation of biofilms by *Yersinia pseudotuberculosis* (*Yptb*) and *Y. pestis* requires the *hmsHFRS* genes, which direct production of a polysaccharide extracellular matrix (Hms-ECM). Despite possessing identical *hmsHFRS* sequences, *Yptb* produces much less Hms-ECM than *Y. pestis*. The regulatory influences that control *Yptb* Hms-ECM production and biofilm formation are not fully understood. In this study, negative regulators of biofilm production in *Yptb* were identified. Inactivation of the BarA/UvrY two-component system or the CsrB regulatory RNA increased binding of Congo Red dye, which correlates with extracellular polysaccharide production. These mutants also produced biofilms that were substantially more cohesive than the wild type strain. Disruption of *uvrY* was not sufficient for *Yptb* to cause proventricular blockage during infection of *Xenopsylla cheopis* fleas. However, this strain was less acutely toxic toward fleas than wild type *Yptb*. Flow cytometry measurements of lectin binding indicated that *Yptb* BarA/UvrY/CsrB mutants may produce higher levels of other carbohydrates in addition to poly-GlcNAc Hms-ECM. In an effort to characterize the relevant downstream targets of the BarA/UvrY system, we conducted a proteomic analysis to identify proteins with lower abundance in the *csrB*::Tn5 mutant strain. Urease subunit proteins were less abundant and urease enzymatic activity was lower, which likely reduced toxicity toward fleas. Loss of CsrB impacted expression of several potential regulatory proteins that may influence biofilms, including the RcsB regulator. Overexpression of CsrB did not alter the Congo-red binding phenotype of an *rcsB*::Tn5 mutant, suggesting that the effect of CsrB on biofilms may require RcsB. These results underscore the regulatory and compositional differences between *Yptb* and *Y. pestis* biofilms. By activating CsrB expression, the *Yptb* BarA/UvrY two-component system has pleiotropic effects that impact biofilm production and stability.

## Introduction

Like many bacteria, *Yersinia pseudotuberculosis* (*Yptb*) in varied environments such as soil and water face temperature extremes, desiccation, nutrient deprivation, or nematode predation, and their survival is enhanced by efficient biofilm production. Transmission of *Yersinia pestis* by fleas is also significantly influenced by biofilm production. One transmission mechanism exhibited by some fleas requires *Y. pestis* to form biofilm on the spines that line the interior surface of the flea's proventriculus (Hinnebusch et al., 1996; Jarrett et al., 2004). Biofilm formation in both *Yptb* and *Y. pestis* is aided by the *hmsHFRS* gene products, which together direct the synthesis of an extracellular matrix (ECM) containing poly-β-1,6 linked *N*-acetyl-D-glucosamine (β-1,6-GlcNAc) (Bobrov et al., 2008; Erickson et al., 2008; Hinnebusch and Erickson, 2008). HmsR and HmsS are inner-membrane proteins that may assemble polymers from UDP-*N*-acetylglucosamine in the cytoplasm. The polymer is likely deacetylated by HmsF prior to export and transport through the outer membrane porin HmsH. Hms-ECM production by *Yptb* and *Y. pestis* can be visualized as Congo red binding (pigmentation) on agar plates. All four of the *hmsHFRS* gene products are required for pigmentation and biofilm, and inactivation of the periplasmic, deacetylase, and glycosyl transferase domains of HmsH, F, and R, respectively, reduces Congo red binding and *in vitro* biofilm formation (Forman et al., 2006).

Recent efforts have focused on understanding how biofilm production in *Y. pestis* is regulated. Multiple regulatory influences of the Hms-ECM in *Y. pestis* have been identified including temperature (Perry et al., 2004), polyamines (Wortham et al., 2010), and the second messenger cyclic-diguanylate (c-di-GMP) (Kirillina et al., 2004; Bobrov et al., 2011). Hms-ECM is produced at growth temperatures ≤26°C (Perry et al., 1990; Hinnebusch et al., 1996; Darby et al., 2002; Jarrett et al., 2004). Temperature regulation is achieved in part by the reduced translation or stability of HmsH, HmsR and the diguanylate cyclase HmsT (Perry et al., 2004) at temperatures above 28°C. High levels of cyclic diguanylate in the cell may increase the glycosyl transferase activity of HmsR (Bobrov et al., 2008). *Y. pestis* contains an additional diguanylate cyclase (HmsD) that enhances biofilm formation in the flea digestive tract (Bobrov et al., 2011; Sun et al., 2011).

Less is known about the regulation of biofilm in *Yptb*. Although *Yptb*, like *Y. pestis*, forms Hms-dependent biofilms *in vitro* and on the outer mouthparts of *Caenorhabditis elegans* nematodes (Darby et al., 2002; Joshua et al., 2003), the majority of *Yptb* strains do not form pigmented colonies on Congo-red agar. Additionally, *Yptb* never forms biofilm on the flea proventriculus to cause blockage of the digestive tract, even though it can colonize the flea midgut (Erickson et al., 2006). Compositional changes in the ECM of *Y. pestis* and *Yptb* may contribute to this difference but it is now clear that during the evolution of *Y. pestis*, mutations in genes that affect the regulation of Hms protein synthesis or production and stability of the ECM have been selected. Functional regulatory mechanisms that repress Hms-dependent ECM production in *Yptb* such as the RcsA transcriptional regulator (Sun et al., 2008; Guo et al., 2015), the NghA glycosyl hydrolase (Erickson et al., 2008), and additional enzymes that produce or degrade cyclic diguanylate (Bobrov et al., 2011) have been lost or altered during the emergence of *Y. pestis*, which have enhanced its biofilm production within fleas.

In this study we sought to identify additional regulatory mechanisms that repress production of extracellular polysaccharides in *Yptb*. A screen for mutants with enhanced Congo Red pigmentation phenotypes suggested a role for the BarA/UvrY two-component regulatory system. We tested the effect of *barA, uvrY* and *csrB* mutation in Congo Red binding and biofilm stability. We sought to correlate the changes we observed in biofilm stability and Congo-red binding with production of specific carbohydrates using fluorescently-labeled lectins and flow cytometry. Finally, we employed a proteomic approach to identify multiple downstream targets of the CsrB regulatory RNA that could influence biofilm formation.

## Materials and methods

### Bacterial strains and growth conditions

*Y. pseudotuberculosis* strain IP32953 and *Y. pestis* KIM6+ were routinely grown at 28°C in Terrific broth (TB) or at 21°C in 1% heart infusion broth supplemented with 0.2% galactose (HIG). The transposon mutants in strain IP32953 were generated using the pRL27 Tn5 donor plasmid as described previously (Erickson et al., 2016). The mutants were plated onto HIG agar containing 0.01% Congo-Red dye. After growth on Congo-red agar plates for 48 h, mutants with increased pigmentation were selected and the location of their transposon insertions determined using arbitrary PCR and sequencing as previously described (Erickson et al., 2016).

To complement the transposon insertion mutants, copies of *barA, uvrY, rcsA*, and *rcsB* were amplified by PCR from IP32953 genomic DNA (all primers are listed in Supplementary Table 1) and inserted into pJET1.2. The CsrB expression plasmid contains the *csrB* sequence in plasmid pACYC184 and was generously supplied by Petra Dersch. The *csrA* gene was also overexpressed by cloning into pJET1.2. Plasmids were created in *E. coli* strain DH5α and transferred to *Yptb* by electroporation.

### Biofilm tests

Liquid biofilms were grown in 24-well plates in 1 ml of HIG broth. The plates were incubated at 21°C for 48 h with shaking at 100 rpm. The unattached cells were removed and the wells were gently washed with 1 ml water, and then the plates were dried for 30 min at 80°C. The attached cells were stained for 20 min with 0.1% crystal violet and rinsed 3 times with water. The bound dye was solubilized by adding 1 ml DMSO and then the absorbance was measured at 590 nm.

To measure biofilm stability, biofilms were grown on 0.2 μm polycarbonate filters (25 mm) placed on HIG agar plates. After growth in liquid media overnight, 5 μl of saturated culture was added to the filters and incubated for 72 h. The filters containing the biofilms were transferred to 50 ml conical tubes containing 40 ml of phosphate-buffered saline (PBS). The tubes were placed horizontally and shaken at 100 rpm for 1 h. The density of the cells that were dislodged during this time was measured by spectrophotometry at 600 nm. The tubes were then vortexed vigorously for 5 min to suspend all of the bacteria that were present on the filters, and the density of the total population was measured. The absorbance of the biofilm cells that were initially dislodged was divided by the absorbance after vortexing to calculate the percent disruption after 1 h.

### Lectin binding flow cytometry

Bacteria were grown on HIG agar plates for 24 h. Single colonies were washed and resuspended in 300 μl of 20 mM HEPES buffer containing 0.1 mM each of MgCl_2_, CaCl_2_, MnCl_2_. Washed bacteria were diluted 1:10 into HEPES buffer containing 0.2 mg/ml FITC-labeled lectin (Wheat germ agglutinin or *Ulex europaeus* agglutinin, Sigma). Samples were incubated on ice in the dark for 30 min and then washed twice in HEPES buffer and finally resuspended in HEPES containing 1% formaldehyde. The fluorescence of individual bacterial cells was measured using a BD FACSCanto II flow cytometer. Negative control samples contained bacteria with no lectin added. The specificity of the lectin binding was assessed by adding 10 mM N,-N′-Diacetylchitobiose or L-(-)-Fucose (Sigma) to the WGA or UEA reactions, respectively.

### Flea infections

*X. cheopis* were orally infected with *Yptb* through shaved mouse skins using a membrane feeder apparatus and monitored for survival and proventricular blockage as previously described (Erickson et al., 2006, 2007; Zhou et al., 2012). For each experiment, ~200 fleas were allowed to feed on 5 ml of heparinized human blood containing 5–8 × 10^8^ colony-forming units (CFU) *Yptb* wild type IP32953 or *uvrY*::Tn5 per ml. Feedings lasted 1 h, and the blood was kept at 37°C during the feeding. Control fleas were fed under the same conditions, on the same blood source but without bacteria. All of the female fleas that ingested blood meals were separated and monitored for 24 h to determine acute toxicity. All of the male fleas were visually inspected for development of proventricular blockage characterized by appearance of fresh blood in the esophagus but not the midgut during twice-weekly feedings for 4 weeks. After 28 days, 20 fleas from each group were homogenized and plated on TB agar containing 1 μg/ml irgasan, 0.5 μg/ml crystal violet, and 1 mg/ml bile salts to determine the proportion of fleas that remained infected throughout the experiment.

### Proteome samples

Samples for label-free proteomic analysis of wild-type and *csrB*::Tn5 mutant were prepared using a filter aided sample preparation (FASP) protocol as described previously (Wiśniewski et al., 2009). Bacteria were grown for 24 h at 21°C on solid HIG media and then washed and frozen. Frozen cell pellets were dropped into 95°C SDT lysis buffer (4% sodium dodecyl sulfate, 100 mM Tris-HCl, 0.1M dithiothreitol) and incubated at 95°C for 10 m. Cell suspensions were sonicated for 30 s (3 Watts of power) and incubated for an additional 10 m at 95°C. Cell extracts were clarified, and clarified lysate was mixed with UA (8 M urea, 100 mM Tris-HCl pH 8.5) in a ratio of 1:5 and applied to Vivacon-500 30,000 MWCO filters (Sartorius, Göttingen, Germany). The filters were washed twice with 100 μL buffer UA. Filters were then incubated 20 min in the dark with 100 μL buffer UA amended with 50 mM iodoacetamide and washed twice with UA and twice with 100 μL 50 mM ammonium bicarbonate. Samples were then digested with trypsin (1 μg proteomic grade trypsin added to 40 μg of total protein in 50 mM ammonium bicarbonate). The filters were incubated at 37°C for 16 h. Peptides were eluted with 50 mM ammonium bicarbonate and acidified to 1% formic acid.

### Ultra-performance liquid chromatography coupled with mass spectrometry (UPLC-MS/MS) and data analysis

Tryptic peptides from the wild-type and the mutant *csrB*::Tn5 were analyzed with reversed phase chromatography (RPC) on an Ultra Performance Liquid Chromatography (UPLC) Eksigent NanoLC system coupled with an LTQ Orbitrap XL mass spectrometer (Thermo Scientific) equipped with an electrospray ionization source (ESI). Specifically, 4 μL of digested proteins were loaded on to a C18-trap column (Waters Corporation), and were desalted with 97% mobile phase A (0.1% formic acid in H_2_O, Optima) and 3% of mobile phase B (0.1% formic acid in CH_3_CN, Optima) at a flow rate of 4 μL/min for 10 min. A linear gradient at a flow rate of 325 nL/min changing from 3 to 35% of mobile phase B was then applied to load the peptides onto an online Waters Peptide Separation Technology C18 column in 90 min, and further sprayed into LTQ Orbitrap XL MS. The mass spectrometer was operated in the positive ion mode, and MS1 was analyzed at a resolution of 60,000 at m/z 400. MS/MS fragmentation analysis was performed on the top 10 most abundant MS1 precursor ions with collision-induced dissociation at a collision energy of 35 V. The online XCalibur software (Thermo Fisher Scientific) was used for data collection. MaxQuant software (Max Plank Institute of Biochemistry) was utilized for MS-based proteomics data analysis, e.g., protein identification and quantification. A *Yptb* IP32953 FASTA file downloaded from Uniprot (The UniProt, 2017) was applied for protein identification with 1% false discovery rate (FDR). A label free quantification (LFQ) approach was utilized for screening differently expressed proteins, with LFQ min. ratio count set as 2.

### Urease assay

Urease activity was quantified as previously described (Onal Okyay and Frigi Rodrigues, 2013) with modifications. Bacteria grown in HIG were pelleted, washed, and resuspended in PBS to an absorbance (600 nm) of 1.0. Stuart's broth (20 g/L urea, 0.1 g/L yeast extract, 0.095 g/L disodium phosphate, 0.091 g/L monopotassium phosphate, 0.01 g/L phenol red) was used as the assay medium. Bacterial suspensions (20 μl) were added to 1 ml Stuart's broth and incubated at 21°C. Every 20 min for 4 h, the absorbance at 560 nm was measured, which corresponds to an increase in pH due to the hydrolysis of urea detected by the color change from yellow to pink of the phenol red indicator.

## Results

### *Yptb* mutants that form pigmented colonies on congo-red agar

To identify genes that repress the formation of the Hms-dependent ECM, we used random Tn5 mutagenesis of *Yptb* strain IP32953, which normally forms white or very light-pink colonies on Congo-red agar. From a screen of ~15,000 colonies from four different mutagenesis experiments, we isolated 35 mutants that consistently produced colonies that were noticeably darker than the wild-type when grown at 21°C on Congo-red agar. We identified the transposon insertion sites by sequencing arbitrary PCR products and comparing these sequences to the *Yptb* IP32953 genome. The locations of the insertions are listed in Table 1. As expected, we obtained insertion mutants in several known negative regulators of Hms expression, including the *rcsA* and *rcsB* transcriptional regulators of the Rcs phosphorelay system and the phosphodiesterase *hmsP*. Insertions were also found in or near genes not previously reported to affect Hms function, including transcriptional regulator *nhaR*, the chaperone *djlA*, and the DNA-binding protein *dps*. NhaR is a transcriptional activator of the *E. coli pgaABCD* genes, which are orthologs of the *hmsHFRS* operon (Goller et al., 2006; Cerca and Jefferson, 2008). DjlA is a member of the DnaJ family of inner membrane co-chaperone proteins, whose overexpression leads to increased production of *E. coli* colanic acid capsule via activation of the Rcs phosphorelay system (Clarke et al., 1997; Chen et al., 2001; Shiba et al., 2006).

**Table 1:** *Yptb* strain IP32953 Tn5 insertion mutants with increased Congo-red binding.

**Tn5 insertion location**	**YPTB ORF**	**Name/function**
66366	YPTB0055	hldD ADP-L-glycero-D-manno-heptose-6-epimerase
67035	YPTB0055	hldD ADP-L-glycero-D-manno-heptose-6-epimerase
233986	YPTB0194	uvrD helicase
420511	YPTB0355	Putative phage inhibition, colistin resistance protein
683674	YPTB0580	Putative Na+ dependent nucleoside transporter-family protein
702455	YPTB0594	Putative Ca++ transporting P-type ATPase
729594	YPTB0614	nhaR transcriptional activator protein
757451		intergenic, adjacent to djlA colanic acid regulator
903733	YPTB0750	barA sensor kinase
1211890	YPTB1009	gmd; GDP-mannose dehydratase
1212962	YPTB1010	fcl; GDP-fucose synthetase
1213231	YPTB1010	fcl; GDP-fucose synthetase
1504191	YPTB1258	rcsB; regulator of colanic acid
2093492	YPTB1735	uvrY response regulator
2093513	YPTB1735	uvrY response regulator
2093729	YPTB1735	uvrY response regulator
2093834	YPTB1735	uvrY response regulator
2093927	YPTB1735	uvrY response regulator
2757792	YPTB2335	ihf; integration host factor alpha subunit
2797859	YPTB2373	Putative fimbrial chaperone protein
2803602	YPTB2377	Putative solute/DNA competence effector
2931611	YPTB2486	rcsA; regulator of colanic acid
2931807	YPTB2486	rcsA; regulator of colanic acid
2931914	YPTB2486	rcsA; regulator of colanic acid
3012673	YPTB2546	dps; DNA binding during starvation
3013236	YPTB2546	dps; DNA binding during starvation
3458503	YPTB2924	seqA; negative regulator of cell division
3467542	YPTB2935	Putative periplasmic transport protein
3550221		csrB regulatory RNA
3550315		csrB regulatory RNA
3550460		csrB regulatory RNA
3819095	YPTB3244	Hypothetical protein
3819394	YPTB3244	Hypothetical protein
4450576	YPTB3750	Shikimate kinase I
4581248	YPTB3836	hmsP phosphodiesterase

We obtained several independent insertions in the genes encoding the BarA sensor kinase, the UvrY response regulator, and the CsrB small RNA that resulted in increased pigmentation on Congo-red plates. The CsrA regulatory protein is an RNA-binding protein that primarily represses translation of target mRNA molecules by binding at sites containing A/UCANGGANGU/A motifs, often at or near the Shine-Dalgarno sequence (Timmermans and Van Melderen, 2010). The *pgaABCD* transcript in *E. coli* contains three CsrA-binding sites in its 5′ untranslated region (Wang et al., 2005), and mutants that lack CsrA produce higher than normal levels of β-1,6-GlcNAc polysaccharide. CsrB and CsrC are small RNAs with high affinity for CsrA that when expressed can relieve CsrA-mediated inhibition of translation. In response to accumulation of by-products of glycolysis such as acetate and formate (Chavez et al., 2010), BarA phosphorylates UvrY, which then activates transcription of genes including the CsrB regulatory RNA (Suzuki et al., 2002). Thus, mutations in *barA, uvrY*, or *csrB* decrease β-1,6-GlcNAc production in *E. coli*. Our transposon mutagenesis results suggested that the opposite is true in *Yptb*, which led us to focus our investigation on this regulatory system.

### The role of Bara/UvrY/CsrB on biofilm stability and extracellular polysaccharide production

In order to verify that the transposon insertions did not affect expression of other genes, we complemented *uvrY*::Tn5 and *barA*::Tn5 strains with plasmids containing functional *barA* or *uvrY* genes. Overexpression of *barA* restored the wild-type phenotype (non-pigmented colonies) in the *barA*::Tn5 background. However, overexpression of *uvrY* in this strain resulted in an intermediate pigmentation phenotype, suggesting that phosphorylation of UvrY by BarA is required for UvrY to fully activate the relevant downstream targets. Overexpression of CsrB in both the *barA::*Tn5 and *uvrY::*Tn5 strains restored the non-pigmented phenotype, suggesting that the regulatory effect of BarA/UvrY occurs largely through CsrB and its regulation of CsrA. When we overexpressed the CsrA regulatory protein in wild type *Yptb* the cells were highly pigmented, but they grew more slowly and many non-pigmented colonies appeared in the population (data not shown) suggesting a toxic effect. Thus, the effect of the BarA/UvrY/CsrB system most likely affects pigmentation by regulating CsrA activity.

**Figure 1:**
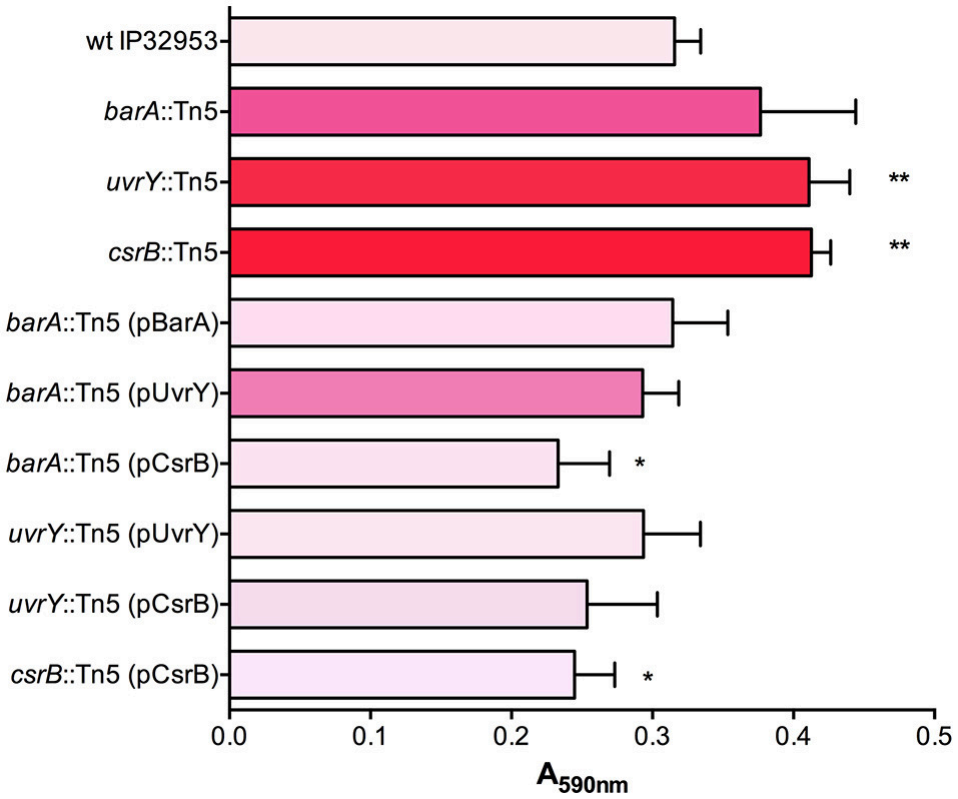
BarA, UvrY, and CsrB negatively regulate biofilm production in *Yptb* IP32953. Congo-red binding phenotypes were qualitatively assessed and categorized (light pink indicates little to no binding and red indicates strongly pigmented colonies). Biofilm attachment after 48 h to polystyrene plates was quantified by crystal violet staining (A_590nm_). Results are means and standard deviations for a representative experiment (*n* = 3) that was performed three times. One-way ANOVA analysis was performed with Tukey's correction for multiple comparisons and strains significantly different from the wild type strain are indicated (*adjusted *P*-value < 0.05, ** < 0.01).

To define the role of this system in biofilm production, we first tested the ability of *Yptb* mutants lacking BarA or UvrY to form biofilms in 24-well polystyrene plates (Figure 1). The *uvrY::*Tn5 and *csrB::*Tn5 strains formed slightly thicker biofilms than the wild type, whereas the *barA* mutant strain was not significantly different from wild type in this assay. Similar to the Congo-red binding, overexpression of CsrB was sufficient to reduce biofilm production in the *barA*::Tn5 and *uvrY::*Tn5 mutants.

**Figure 2:**
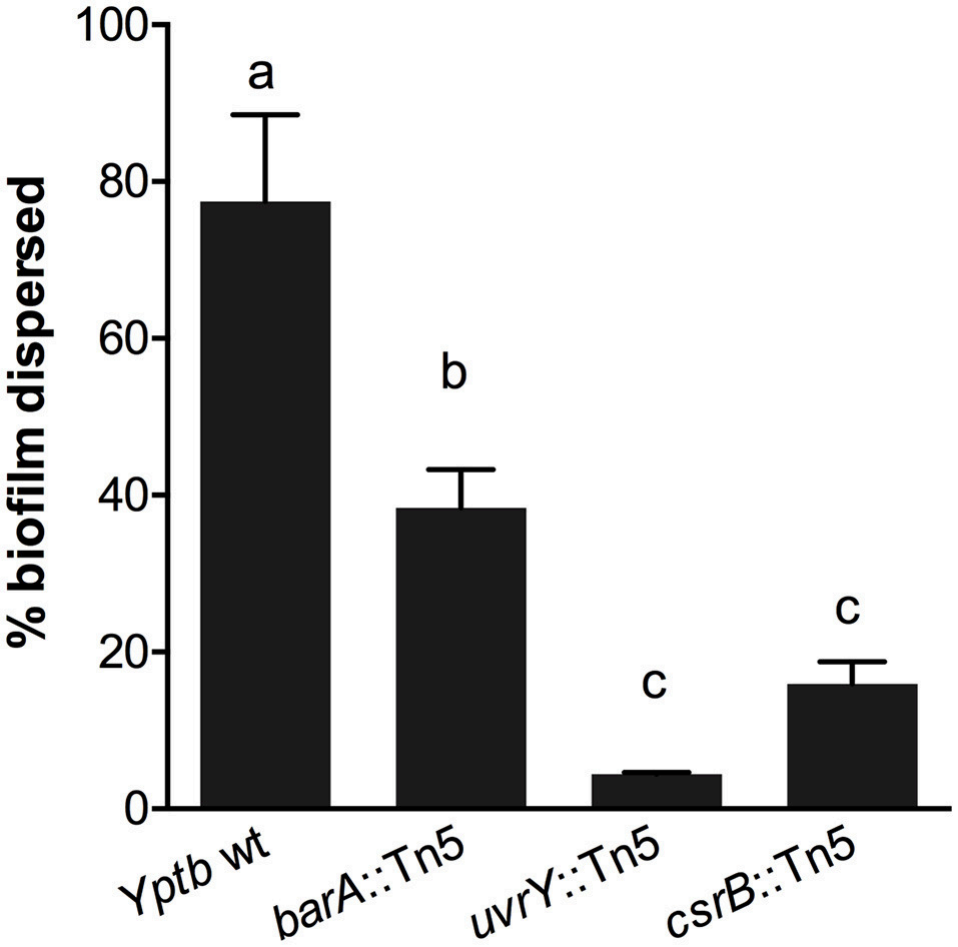
Loss of *uvrY* or *csrB* increases the cohesiveness of *Yptb* biofilms. Biofilms of each strain were grown on polycarbonate filters and then transferred to saline and agitated for 1 h. The proportion of the biofilm that was disrupted by the agitation was measured. Results are means and standard deviations of three independent experiments. One-way ANOVA analysis was performed with Tukey's correction for multiple comparisons. Columns with the same letter (a, b, c) are not significantly different from each other (95% confidence interval).

We used an additional assay specifically to investigate the cohesiveness of the biofilms formed by these strains. We allowed biofilms to form on polycarbonate filters and then measured the tendency of the biofilm to become dislodged and cells to become suspended in solution. The biofilms were placed in tubes with saline and agitated. The proportion of the biofilm that was dislodged and suspended in the solution was measured after a 1-h period (Figure 2). This assay showed that the wild type biofilms were easily disrupted, whereas the *csrB*::Tn5 and *uvrY*::Tn5 biofilms strongly adhered together. The *barA*::Tn5 biofilms were less cohesive than the *uvrY*::Tn5 or *csrB*::Tn5 biofilms but more than the wild type, similar to the Congo-red binding results.

**Figure 3:**
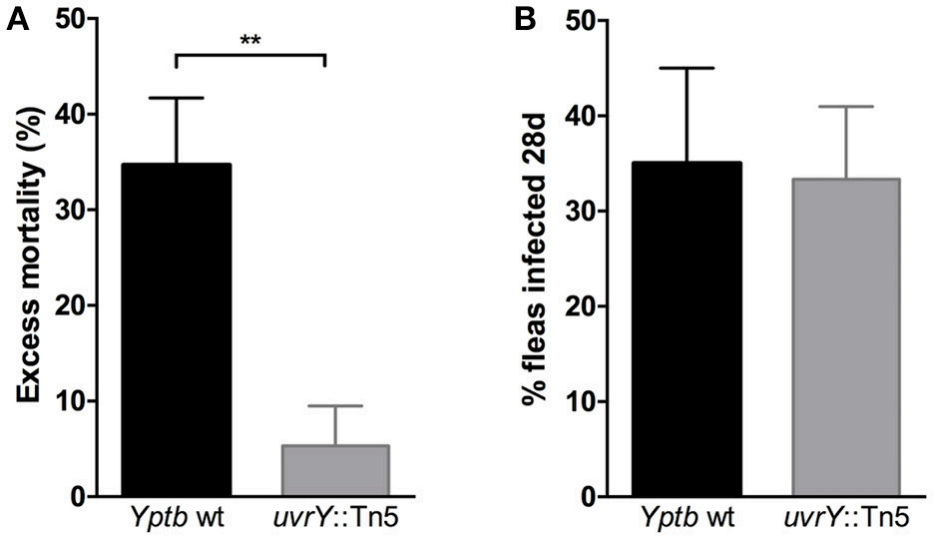
Loss of *uvrY* does not affect *Yptb* colonization or blockage due to biofilm but does reduce acute oral toxicity toward *X. cheopis* fleas. Fleas were fed blood meals containing 10^8^ CFU/ml wild type or *uvrY*::Tn5 mutant strain. **(A)** The proportion of fed fleas that died within 24 h of infection was significantly less for the *uvrY*::Tn5 mutant compared to the wild type strain (**p < 0.01 by unpaired *T*-test) in three independent feeding experiments. **(B)** Proportions of fleas that remained infected with the *uvrY*::Tn5 mutant or wild type strain after 28 days. No signs of proventricular blockage were observed in any *Yptb*-infected fleas (wild type or mutant) during twice-weekly feedings.

Since the *uvrY*::Tn5 mutant produces strongly cohesive biofilms *in vitro*, we considered whether this strain could form biofilms in fleas and cause proventricular blockage similar to *Y. pestis*, or to *Yptb* mutants that lack *rcsA, hmsT* and *hmsD* (Sun et al., 2014). We infected *Xenopsylla cheopis* fleas with wild type *Yptb* or the *uvrY*::Tn5 mutant, and monitored the fleas for 28 days for signs of proventricular blockage. Although both strains were able to colonize and maintain stable infections, none of the fleas were observed to develop blockage over this period. However, more fleas infected with the mutant strain survived the first 24 h after infection than with the wild type strain (Figure 3), suggesting that UvrY may regulate acute toxicity of *Yptb* within the flea digestive tract.

**Figure 4:**
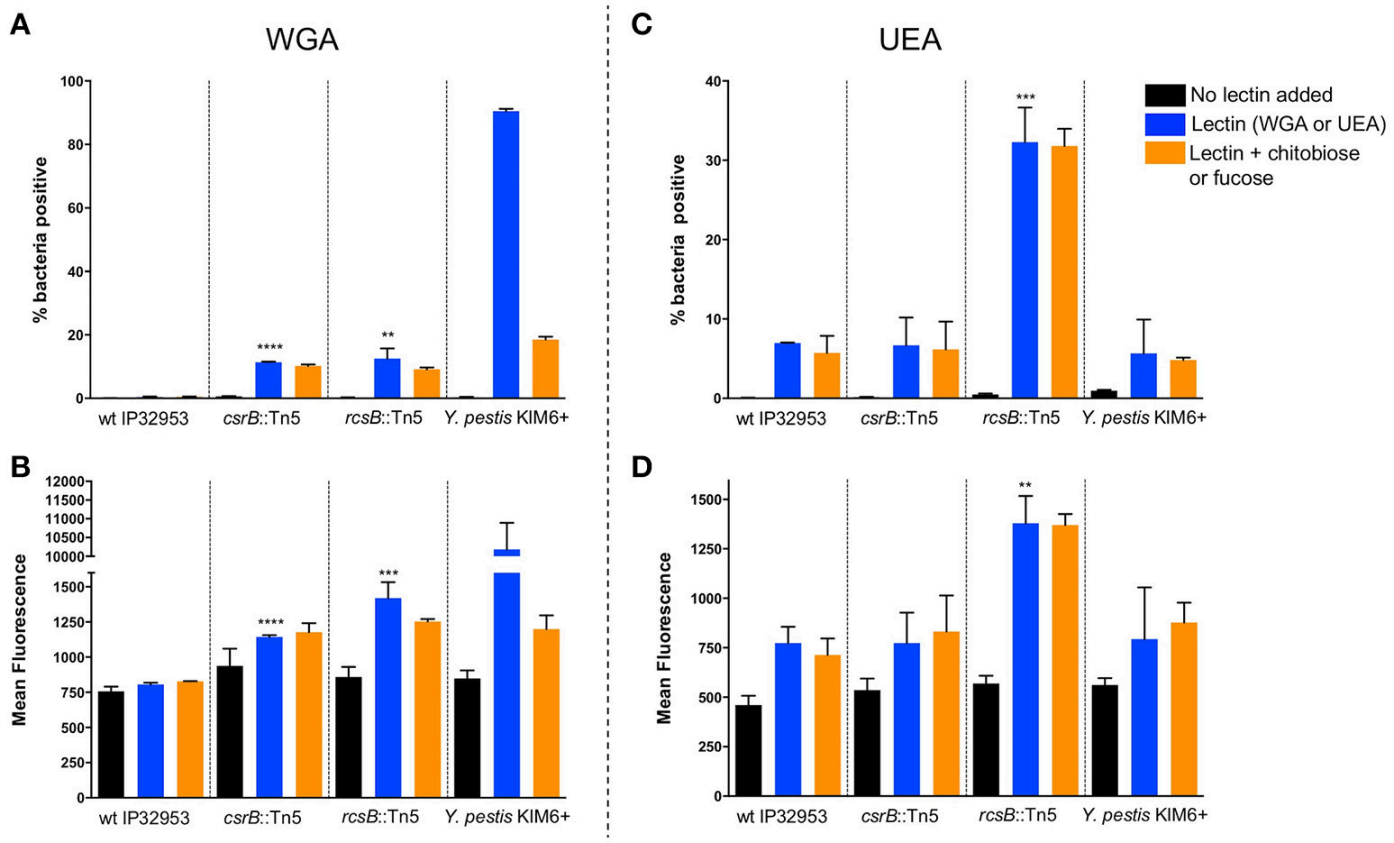
Loss of *rcsB* and *csrB* alters lectin binding to bacterial surfaces. Bacteria were incubated with FITC-labeled WGA **(A,B)** or UEA **(C,D)** lectins. Flow cytometry was used to measure the proportion of bacteria with any detectable binding **(A,C)** or the mean fluorescence of the population **(B,D)**. For each strain that was tested, a control with no lectin was included to establish baseline fluorescence, as well as samples with competing sugars (chitobiose or fucose). Results are means and standard deviations for a representative experiment that was performed four times. To see if the *csrB*::Tn5 or *rcsB*::Tn5 mutants bound lectin differently than the wild type strain, one-way ANOVA analysis was performed with Tukey's correction for multiple comparisons using the lectin-treated samples (blue bars). The *csrB*::Tn5 and *rcsB*::Tn5 mutants bound significantly more WGA than the wild type strain, but only the *rcsB*::Tn5 mutant bound more UEA than wild type. *Y. pestis* binding was tested as a control, and it bound to WGA far more efficiently than *Yptb* (**p < 0.01, ***p < 0.001, ****p < 0.0001).

Pigmentation of *Yersinia* colonies on Congo-red agar is correlated with Hms-ECM production, but additional proteins and carbohydrates are also able to bind the dye. To more specifically assess the surface carbohydrate differences in these strains, we employed fluorescently-labeled lectins with affinity to specific sugars. Flow cytometry was used to measure the fluorescence of individual bacteria after labeling with wheat-germ agglutinin (WGA, Figures 4A,B), which binds to dimers or trimers of *N*-acetylglucosamine, or to *Ulex europaeus* agglutinin (UEA-1, Figures 4C,D), which binds to α-linked fucose residues. We measured the percentage of bacteria within each sample with detectable lectin binding (% positive, Figures 4A,C) as well as the mean fluorescence of the entire population (Figures 4B,D). We found that the binding of WGA to *Y. pestis* KIM6+ was very high, and this binding was strongly inhibited by addition of competing N-acetyl-chitobiose. UEA-1 bound only modestly to *Y. pestis*, consistent with the majority of the extracellular carbohydrate consisting of Hms-ECM. Surprisingly, the wild type *Yptb* strain did not bind detectably to WGA and only moderately to UEA-1. This binding was not inhibited by the addition of competing chitobiose (for WGA) or fucose (for UEA-1), suggesting the possibility of secondary or tertiary sugar preferences for lectin binding to these bacteria. The *csrB*::Tn5 mutant strain bound more WGA lectin than the wild type strain, which is consistent with greater Hms-ECM production, but again the addition of competing chitobiose did not reduce lectin binding. Surprisingly, the *rcsB*::Tn5 mutant strain, which is known to express high levels of cyclic diguanylate and Hms-ECM, bound more strongly to UEA-1 than to WGA. These results suggest differences in the extracellular matrix between *Y. pestis* and *Yptb*.

### Proteomic investigation of BarA/UvrY regulation through CsrB

Phosphorylated UvrY upregulates CsrB expression in *E. coli* and *Yersinia*. Multiple copies of CsrA protein are bound by CsrB RNA, thus preventing it from binding its target mRNAs. In *E. coli*, CsrA binds to *pgaABCD* transcripts to repress their translation. CsrA is most often a translational repressor, but enhances translation of some target mRNAs (Potts et al., 2017). Unlike the *pgaABCD* upstream region, no obvious CsrA binding sites were identified within the coding sequences or in the 500 bp region immediately upstream of the *hmsH* start codon. We therefore considered whether the CsrA protein of *Yptb* could instead repress other negative regulators of Hms activity. We applied a proteomic approach on whole cell lysates prepared from wild type *Yptb* strain and the *csrB*::Tn5 mutant using LC-MS/MS analysis and mapping the peptide fragments to the *Yptb* IP32953 database. We selected proteins that were at least 1.5-fold changed in abundance between the two strains (Supplementary Table 2). As expected, the vast majority of the differentially expressed proteins were less abundant in the *csrB* mutant, consistent with the role of CsrA protein as mainly a repressor of translation.

The global changes in transcript abundance in a *Yptb* strain YPIII *csrA* mutant were recently reported by Bücker et al. (2014). Many of the genes that are upregulated in their study showed lower protein levels in this analysis of strain IP32953 *csrB*::Tn5 mutant. The RovA (SlyA) protein is known to be repressed by CsrA (Heroven et al., 2008), and our data indicated a 28-fold decrease in RovA abundance. RovA is controlled by CsrA through the RovM protein, which is activated by CsrA (Heroven et al., 2008). RovM peptides were not detected among the wild type samples, but were in the *csrB*::Tn5 mutant (Supplementary Table 2). Several additional regulatory proteins that could regulate biofilm production were repressed in the *csrB*::Tn5 mutant strain. Nucleoid-associated proteins, including Integration Host Factor alpha (IHFα, 11.8-fold reduction) and histone-like nucleoid structuring protein (H-NS, 24-fold reduction) function to silence and activate gene expression on a global scale (Dillon and Dorman, 2010). IHFα was also identified in our initial transposon screen for mutants that exhibit enhanced Congo-red binding (Table 1). H-NS represses expression of horizontally acquired genes (Lucchini et al., 2006), and the *hmsHFRS* operon is contained within the mobile *Yersinia* high-pathogenicity island (Lillard et al., 1997). H-NS also represses expression of *pgaABCD* and biofilm production in *Actinobacillus pleuropneumoniae* (Bossé et al., 2010). H-NS is believed to be essential for *Yersinia* survival (Ellison and Miller, 2006), so we were unlikely to identify it in our transposon screen.

**Figure 5:**
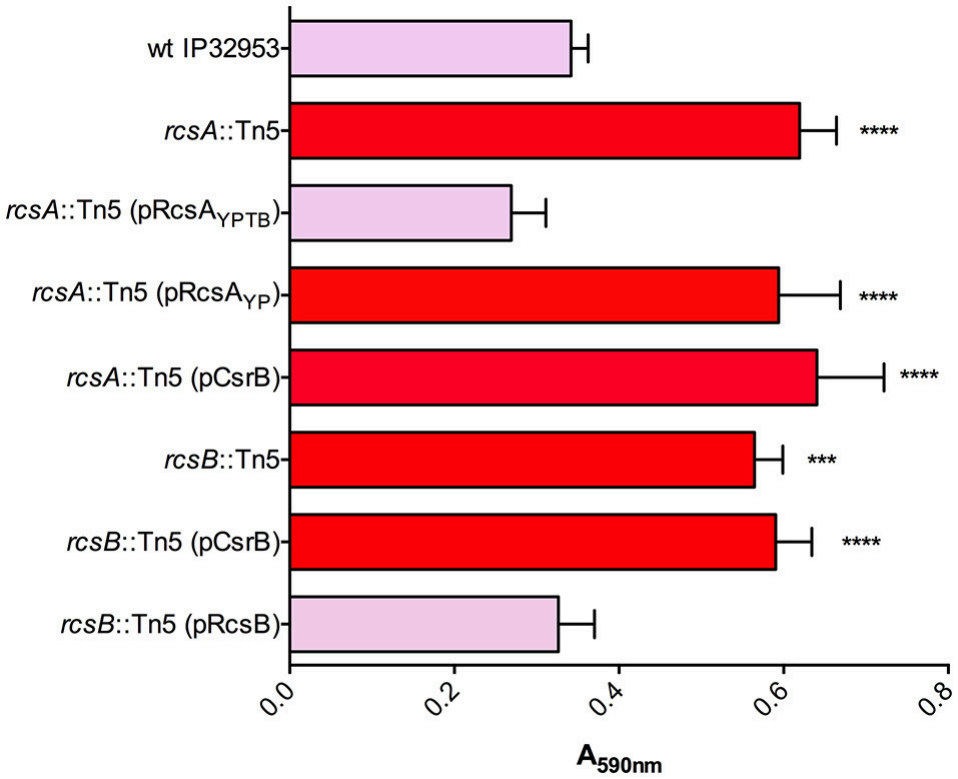
Biofilm and Congo-red binding by *rcsA* and *rcsB* mutants is not affected by overexpression of CsrB. Congo-red binding phenotypes and biofilm attachment in polystyrene plates after 48 h were measured in wild type *Yptb* and in *rcsA*::Tn5 or *rcsB*::Tn5 mutants. The effects of plasmids containing *rcsA* from *Yptb* (pRcsA_YPTB_) or *Y. pestis* (pRcsA_YP_) were determined in the *rcsA*::Tn5 mutant strain. The *rcsB*::Tn5 mutant was complemented with a plasmid containing *rcsB* from *Yptb*. Overexpression of CsrB (pCsrB), which reduced Congo-red binding and biofilm production in *barA*::Tn5 and *uvrY*::Tn5 mutants (Figure 1), was also tested in the *rcsA* and *rcsB* mutants. Results are means and standard deviations for a representative experiment (*n* = 3) that was performed four times. One-way ANOVA analysis was performed with Tukey's correction for multiple comparisons. Values that were significantly different from the wild type are indicated (***adjusted *P*-value < 0.001, **** adjusted *P*-value < 0.0001).

We identified transposon insertions in both *rcsA* and *rcsB* in our screen for Congo-red binding mutants (Table 1). The *Yptb* RcsB transcriptional regulatory protein, together with RcsA, represses transcription of the diguanylate cyclases *hmsT* and *hmsD*, thereby reducing biofilm production. *Y. pestis rcsA* has a frameshift mutation, thus eliminating this repression and enhancing biofilms (Sun et al., 2008, 2014; Sun Y. C. et al., 2012; Guo et al., 2015). To ensure that the transposon insertions in *rcsA* and *rcsB* were not affecting other genes, we complemented both mutants via plasmids containing functional copies of these genes. The *rcsB*::Tn5 mutant was complemented with a plasmid containing *rcsB*. The *rcsA*::Tn5 mutant was able to be complemented via *rcsA* from *Yptb* but not from *Y. pestis*, consistent with the frameshift mutation in *Y. pestis rcsA*. Our proteomic data revealed that *csrB*::Tn5 produced 8-fold less RcsB protein than the wild type strain (Supplementary Table 2). CsrA has not been previously shown to repress expression of RcsB directly or indirectly. To test the relationship between *rcsB* and CsrB further, we overexpressed CsrB in the *Yptb rcsA*::Tn5 or *rcsB*::Tn5 mutant strains (Figure 5). Unlike in the *uvrY*::Tn5 or *barA*::Tn5 mutants, CsrB expression did not reduce Congo-red binding in the *rcsA* or *rcsB* mutants. This suggests the possibility that CsrB competes with *rcsB* transcripts for CsrA binding. The predicted secondary structure of the *rcsB* transcript contains a stem-loop with an exposed GGA motif overlapping the Shine-Delgarno sequence (Supplementary Figure 1), which is characteristic of CsrA targets (Romeo and Babitzke, 2018). Alternatively, the absence of a functional Rcs phosphorelay could overpower the negative regulatory effects of CsrB.

**Figure 6:**
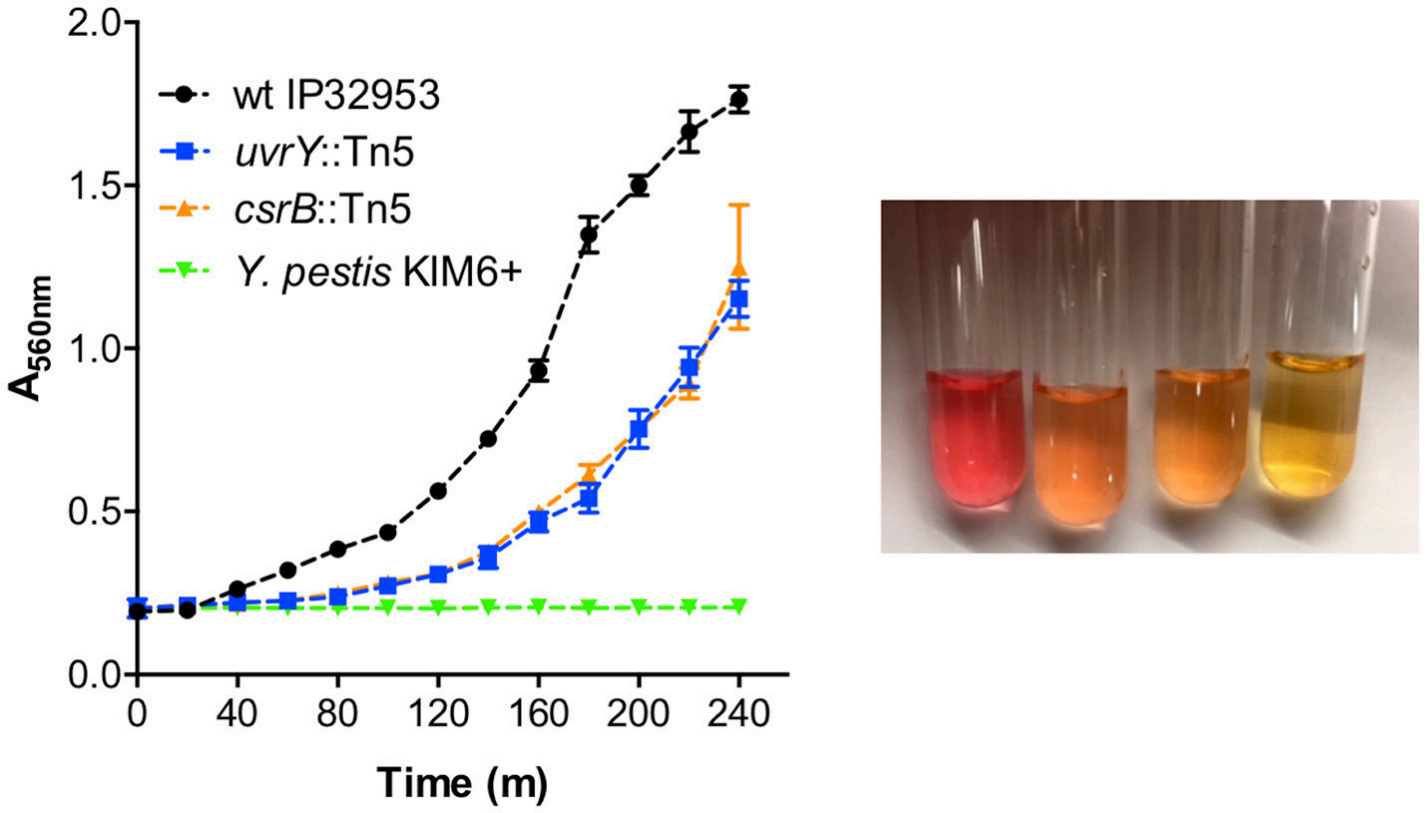
UvrY and CsrB positively regulate urease. Equal numbers of bacteria were suspended in Stuart's broth and pH change due to hydrolysis of urea was detected by color change from yellow to pink (picture from left to right: wild type *Yptb, uvrY*::Tn5, *csrB*::Tn5, and *Y. pestis* after 4 h). Urease was quantified by measuring the absorbance at 560 nm (left panel) every 20 min. Results are means and standard deviations of three independent experiments. Multiple *t*-tests of the data were performed with a false-discovery rate (Q) value set to 1.000%, without assuming a consistent SD. The *uvrY*::Tn5 and *csrB*::Tn5 mutant strains were significantly different (*p* < 0.05) from the wild type strain at every time point after 60 min.

Urease subunit proteins were also substantially reduced in the *csrB*::Tn5 mutant (Supplementary Table 2). The urease structural proteins (UreA 59-fold, UreB 21-fold, UreC 21-fold), and the accessory proteins (UreE 1423-fold, UreF undetected, UreG 45-fold, UreD 71-fold), make up the multimeric urease enzyme. *Y. pestis* lacks a functional UreD subunit, which is sufficient to eliminate urease activity (Sebbane et al., 2001). Unlike *Y. pestis, Yptb* strains are acutely toxic to *X. cheopis* fleas (Erickson et al., 2007), and restoration of urease also restores toxicity to *Y. pestis* (Chouikha and Hinnebusch, 2014). Since the *uvrY*::Tn5 mutant was also less toxic to fleas, we tested whether this strain produces less urease enzyme. The wild type strain produced much more urease than the *uvrY*::Tn5 mutant (Figure 6). The *csrB*::Tn5 mutant also produced much less urease, and no urease activity was detected using *Y. pestis* KIM6+. During the preparation of this manuscript, it was also reported by Dai et al. that CsrA represses urease production in *Yptb* strain YPIII, which is consistent with our results (Dai et al., 2018).

## Discussion

Switching between planktonic and sessile modes of growth is a tightly controlled process involving multiple levels of transcriptional and post-transcriptional regulation. Successful navigation of this transition is critically important to the success of pathogenic yersiniae (Chen et al., 2016). In this study we demonstrated that the BarA/UvrY two component system as well as the small RNA CsrB repress biofilm production in *Yptb*. Disruption of CsrB increased biofilm production while at the same time reducing urease-mediated toxicity toward fleas. Many bacterial pathogens, including *Yersinia* (Bobrov et al., 2011; Zhao et al., 2013; Redelman et al., 2014), exhibit an inverse relationship between biofilm production and acute virulence. The regulatory networks that govern transition between highly toxic phenotypes and the biofilm mode of growth are of interest as possible therapeutic targets. CsrB is known to exert its effects by occupying CsrA proteins, thereby altering expression of CsrA-controlled regulatory proteins and cell-envelope structures that have pleiotropic effects.

Previous work has shown that in *Yptb* strain YPIII, UvrY (and CsrB) expression is normally repressed by the cyclic AMP-Crp complex except during times of nutrient deprivation (Heroven et al., 2012). Starvation conditions would therefore be predicted to promote motility and reduce biofilm formation by reducing the concentration of free CsrA proteins. The downstream targets of CsrA that mediate the biofilm-specific effects are not completely known and may be different between individual strains of *Yptb* or in *Y. pestis*. In *Yptb* strain YPIII, CsrA indirectly activates expression of the RovM transcriptional regulatory protein (Heroven et al., 2008). RovM binds the promoter regions of the *flhDC* flagellar regulatory genes, the *hmsHFRS* operon, and the *rovA* virulence regulator (Heroven et al., 2008; Zhao et al., 2017). Binding of RovM represses *flhDC* and enhances *hmsHFRS* and *rovA* transcription. Others have also shown that CsrA enhances biofilms in *Y. pestis* (Willias et al., 2015). However, loss of CsrA did not change *Y. pestis hmsHFRS, hmsT*, or *hmsP* transcription as occurs in *Yptb*. It will be interesting to determine if RovM activates *hmsHFRS* in *Y. pestis*, or whether RovM is controlled by CsrA in the same fashion as in *Yptb*. We did not identify any transposon insertions in the *rovM* gene (YPTB2588) in our screen for Congo-red binding mutants. This could be because the expression of *rovM* is very low in nutrient-rich conditions such as were used in our study (Heroven and Dersch, 2006). Indeed, RovM protein levels in the wild type strain were below the limit of detection in our proteomic analysis. However, RovM peptides were detected in the *csrB* mutant samples, consistent with CsrA being an activator or RovM expression in *Yptb* strain IP32953. We also did not obtain any transposon insertion mutants in the CsrC regulatory RNA gene in our screen. In contrast to *E. coli*, expression of CsrC is not activated by UvrY in *Yptb* strain YPIII (Heroven et al., 2008). Rather, when CsrB expression is triggered by UvrY, CsrC is repressed by a mechanism that is yet to be determined.

In addition to RovM, our results suggest that other regulatory proteins that control biofilm attachment and stability are under the influence of CsrA. These include the RcsA and RcsB transcriptional regulatory proteins. The Rcs phosphorelay system contains a sensor kinase RcsC that autophosphorylates upon detection of signals that include cellular stress. The phosphate is then transferred through RcsD to RcsB, which then dimerizes and binds to specific promoter sequences. RcsB may also form heterodimers with RcsA, and RcsAB binding represses transcription of diguanylate cyclases *hmsD* and *hmsT*, as well as the *hmsHFRS* operon (Sun Y. C. et al., 2012; Fang et al., 2015). The *rcsA* gene has been disrupted in *Y. pestis*, which was a key event in the evolution of *Y. pestis* transmission by fleas because it greatly enhanced cyclic di-GMP and biofilm production (Sun et al., 2008, 2014). We found that when *rcsA* or *rcsB* were non-functional, overexpression of CsrB did not reduce Congo-red pigmentation or prevent biofilm formation. In our analysis of the *csrB*::Tn5 mutant proteins, RcsA and RcsB proteins were both decreased compared to the wild type strain. These results are suggestive of a possible direct interaction between CsrA and *rcsA* or *rcsB* transcripts, particularly in the case of *rcsB*.

Disruption of *uvrY* or *csrB* had a very strong effect on the cohesiveness of *Yptb* biofilms. We considered that the most likely explanation for this would be increased abundance of extracellular polysaccharide contained in the biofilm matrix, specifically the Hms-ECM in these mutants. *Yersinia* biofilms are often described as bacteria encased in a homopolymer of N-acetyl-D-glucosamine (Hms-ECM) (Zhou and Yang, 2011; Sun F. et al., 2012). Hms-ECM is almost certainly present in *Y. pestis* and *Yptb* biofilms, based on antigenic cross-reactivity of *Y. pestis* biofilms with antibodies directed against purified poly-N-acetylglucosamine (Erickson et al., 2006) and from the extensive genetic data showing the impact of *hmsHFRS* on biofilms in both species. However, our results also suggest that loss of CsrB could control biofilm production by affecting expression of other cell surface molecules. The binding of WGA lectin to *Yptb* mutants that were strongly pigmented on Congo-red agar was far less than what we observed for *Y. pestis*, and binding was barely detectable for the wild type IP32953 strain. Binding of UEA-1 lectin, which is specific for fucose, was increased in the *rcsB* mutant to a greater degree than WGA lectin, which is specific for N-acetylglucosamine. However, these results are complicated by the fact that addition of competing sugars did not reduce lectin binding, except for WGA and *Y. pestis*. The possibility of other proteins, carbohydrates, or nucleic acids within *Yersinia* biofilms deserves further investigation, particularly in *Yptb*. It is likely that Hms-ECM is increased on the surface of *uvrY, csrB* and *rcsB Yptb* mutants, but other macromolecules within the biofilm matrix may interfere with efficient WGA lectin binding. The presence of additional carbohydrates within *Yersinia* biofilms is suggested by previous lectin binding experiments in other *Yptb* strains (Tan and Darby, 2004) as well as genetic studies showing that lipopolysaccharide genes are required for efficient biofilm production in *Y. pestis* (Liu et al., 2016). *Yptb* biofilm extracellular matrix also contains extracellular DNA (Atkinson et al., 2011). The protein component of *Yersinia* biofilms has not been well-studied, but even when lacking the Hms polysaccharide, *Y. pestis* can form biofilm-like aggregates that resist killing by neutrophils (Jarrett et al., 2004). The biochemical nature of the components within these aggregates is unknown, but may suggest that specific *Yersinia* proteins that perform stabilizing and attachment (Fong and Yildiz, 2015) or immune evasion functions within the ECM await discovery.

## Author contributions

JS, JP, and DE conceived and designed the experimental procedures, JS, RS, MS, JC, HK, and DE performed the experiments and carried out data analysis, JS and DE wrote the manuscript.

### Conflict of interest statement

The authors declare that the research was conducted in the absence of any commercial or financial relationships that could be construed as a potential conflict of interest.
