# Supplementary material for: Yersinia pseudotuberculosis BarA-UvrY Two-Component Regulatory System Represses Biofilms via CsrB
Source: Front Cell Infect Microbiol. 2018 Sep 18;8:323. doi: 10.3389/fcimb.2018.00323 (PMC6153318; doi:10.3389/fcimb.2018.00323)
Supplement: Supplementary file 1 [file Table_1.DOCX]

**Supplementary Table 1. PCR primers used in this study**

| Sequence (5’-3’) | Purpose |
| --- | --- |
| taa atg cgc act tcg tac cgc | Complementation of barA |
| ctg cgt gat ggc aaa ccg atc |  |
| caa aca gcc atg aga caa ata | Complementation of uvrY |
| caa cgt ttc acg cac tgc ata |  |
| GCT CGC TTC CGA TAA TTA GGG CGT T | Complementation of rcsA |
| CAC TAC TCA GGC AAG AAA GCA GGT |  |
| GGA AGG TAA CCC ATA ACC ATG AAC | Complementation of rcsB |
| GCA AAA ATT ACA GTT CTT TAT CCA C |  |
