# Supplementary material for: Yersinia pseudotuberculosis BarA-UvrY Two-Component Regulatory System Represses Biofilms via CsrB
Source: Front Cell Infect Microbiol. 2018 Sep 18;8:323. doi: 10.3389/fcimb.2018.00323 (PMC6153318; doi:10.3389/fcimb.2018.00323)
Supplement: Supplementary file 3 [file Data_Sheet_1.PDF]

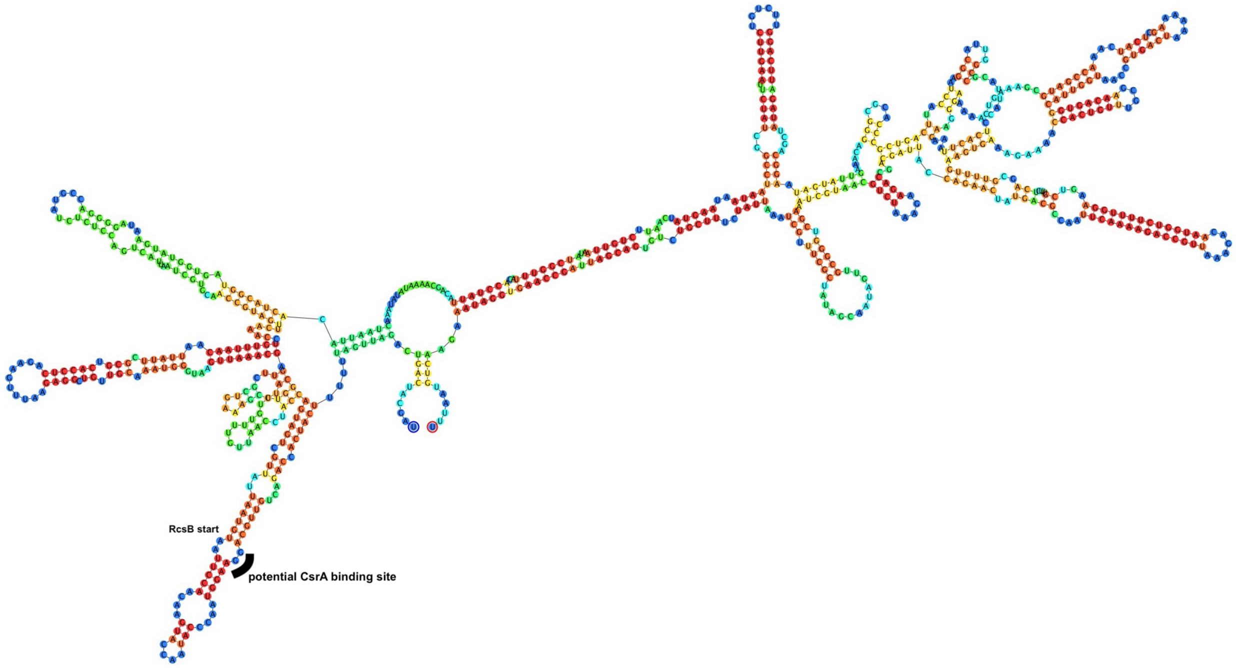

Supplementary Figure 1. Predicted secondary structure of RcsB transcript. The csrB sequence including 60 bases upstream of the start codon was analyzed using the Geneious software package with the RNA fold prediction program. The Andronescu (2007) energy model at 21ºC was used, with red-green-blue representing high-mid-low probability of base pair formation. A potential CsrA-binding site (labeled) was observed in a stem-loop structure 18 bp before the start codon.
